# Synovial sarcoma of the maxillary sinus and chest wall: An exceptional association of two synchronous rare locations, Case report

**DOI:** 10.1016/j.amsu.2021.102523

**Published:** 2021-06-20

**Authors:** Ayoub Sabr, Fatema Ez-zahrae Azami Hassani, Salissou Iro, Faiçal Slimani

**Affiliations:** a Faculty of Medicine and Pharmacy, Hassan II University of Casablanca, B.P, 5696, Casablanca, Morocco; b Oral and Maxillofacial Surgery Department, CHU Ibn Rochd, B.P, 2698, Casablanca, Morocco

**Keywords:** Synovial sarcoma, Maxillary, Chest wall, Chemotherapy, Case report

## Abstract

**Introduction:**

Synovial sarcoma is a malignant tumour of mesenchymal origin with an aggressive character and a rare cervicofacial location. Its management is multidisciplinary in order to improve prognosis and survival.

**Case report:**

We report the case of two rare and synchronous localizations of monophasic synovial sarcoma of the maxilla and chest wall in a 58-year-old woman, whose management was based on exclusive chemotherapy.

**Discussion:**

The cervicofacial location of synovial sarcoma represents 3% of all locations in the body. Its clinical and radiological manifestations are aspecific. Its diagnosis is based on a combination of histological and genetic arguments. Its therapeutic management depends on the tumour stage and prognostic factors.

**Conclusion:**

Early diagnosis of cervicofacial synovial sarcoma allows to avoid a mutilating procedure for the patient and to improve the long-term prognosis by a good control of the tumour.

## Highlights

• Synovial sarcoma is a high-grade histological variety of sarcomas.
• Our case is a perfect example of atypical forms of synovial sarcoma by association of two rare locations that are maxillary bone and chest wall.
• The exact diagnosis is based on a combination of clinical, radiological, histological and genetic arguments.

## 1 Introduction

Synovial sarcoma is a high-grade histological variety of sarcomas [1,12]. It's a rare soft tissue malignant tumor accounting for 5%–10% of all adult soft-tissue sarcomas [3,4,6,9]. it can occur at any age and everywhere in the body [3,9]. the extremities in young adults are the most commonly affected sites [1,5,6], the cervico-facial localization is rare and represents less than 10% of malignant mesenchymal tumours [5,8]. Therapeutic management is multidisciplinary, based on surgery and radiotherapy for localised forms and chemotherapy for extensive forms [8,9].

Our paper reported a case of two rare and synchronous localizations of monophasic synovial sarcoma of the maxilla and chest wall in a 58-year-old woman. The importance of reporting this case lies in the rarity of the facial and chest wall localization of synovial sarcoma, and in our knowledge, it is the first case of synchronous double location of synovial sarcoma interesting two rare locations of this tumor type.

This work has been reported in line with the SCARE 2020 criteria [13].

## 2 Case report

A 58-year-old female patient with high blood pressure on amlodipine 10 mg (1cp/d), presented to our department of maxillofacial surgery in February 2021 for a left maxillary mass that had been evolving for 6 months. The history of the disease goes back to 6 months before his admission with the appearance of a left maxillary tumefaction progressively increasing in volume responsible for homolateral exophthalmos and partial nasal obstruction without any other associated thoracic or abdominal-pelvic discomforts, all evolving in a context of apyrexia and weight loss quantified at 8 Kg in 6 months.

The clinical examination revealed a hard, painless and deep fixed left maxillary mass filling the upper oral vestibule, with no skin or mucosal signs, partial left nasal obstruction and left exophthalmos with no decrease in visual acuity or oculo-motricity disorders. Examination of the lymph nodes did not reveal any palpable cervical or axillary adenopathy. The thoracic and abdominal examination did not find any palpable mass or inflammatory skin signs on the surface.

**Fig. 1:**
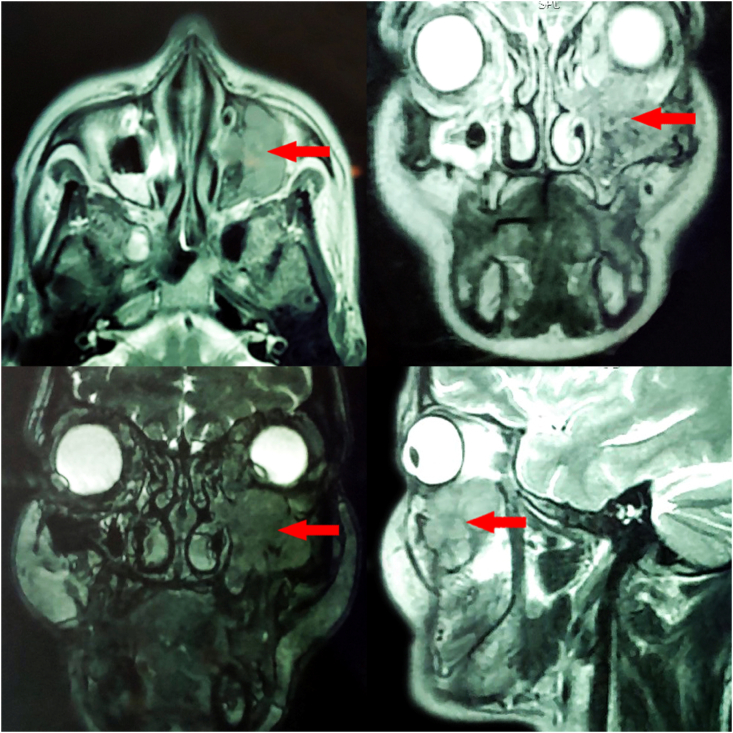
Facial MRI showing a left maxillary sinus tumor in isosignal T1 hypersignal T2 with invasion of the left orbit and nasal cavity.

Facial Magnetic resonance imaging revealed an expansive process with a polylobed contour centred on the left maxillary sinus in T1 isosignal and T2 hypersignal, heterogeneously enhancing after injection of contrast, measuring 32×20×38 mm with extension towards the corresponding jugal soft tissues, the left nasal cavity and the left orbital cavity reaching the contact of the inferior right muscle [Fig. 1].

**Fig. 2:**
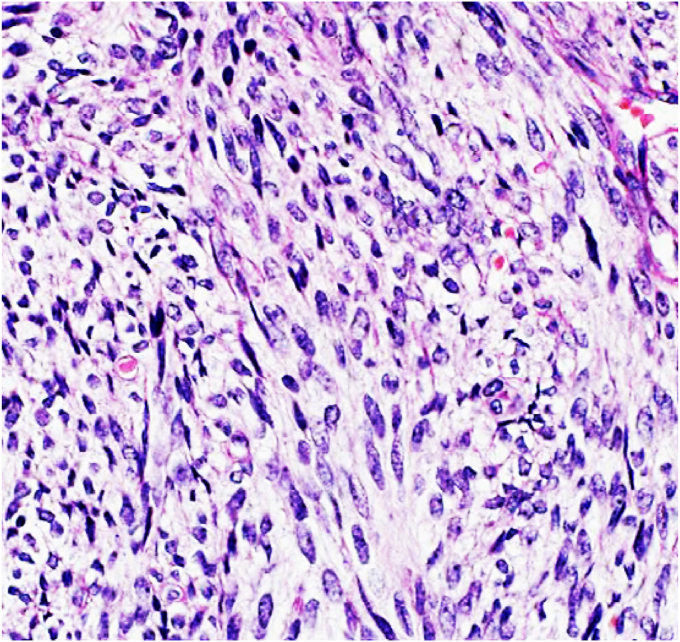
Histological picture of monophasic synovial sarcoma.

A biopsy of the mass was performed under local anesthesia via the left superior vestibular approach and the anatomopathological result came back in favour of a monophasic synovial sarcoma with positivity of the Epithelial Membrane Antigen (EMA) on immunohistochemistry [Fig. 2].

**Fig. 3:**
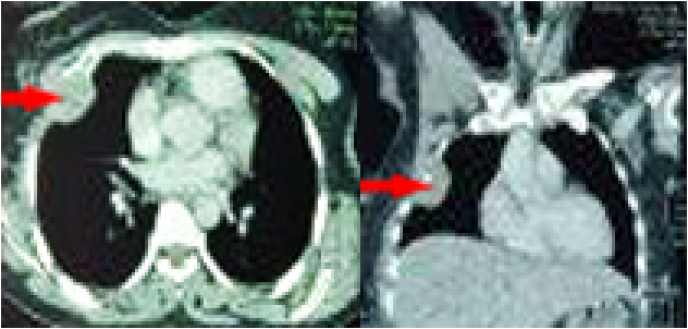
Thoracic CT scan showing a right anterior chest wall tumor.

A thoracic-abdominal-pelvic CT scan was performed to evaluate the extent of the disease, which revealed a tissue mass in the right anterior chest wall measuring 58×43×35 mm with extension to the adjacent lung [Fig. 3]. A surgical biopsy under general anesthesia of the thoracic mass by anterior transcutaneous approach was performed and the anatomopathological result was similar to the left maxillary mass. Given the similar anatomopathological findings of the two maxillary and chest wall masses and the impossibility of identifying the primary location, the diagnosis of synchronous double location of synovial sarcoma was retained.

A multidisciplinary consultation was held to discuss the case and the decision to treat with chemotherapy was indicated. The patient was referred to oncology for curative chemotherapy and then followed as an outpatient at a rate of twice a month for the first three months and then once a month for one year.

## 3 Discussion

Synovial sarcoma is a malignant mesenchymal tumour expanding from pluripotent mesenchymal cells and can develop in all parts of the body [2,10,12]. It is a high histological grade variety of sarcoma and represents the fourth most common entity after malignant fibrous histiocytomas, liposarcomas and rhabdomyosarcomas [1,11]. It is a rare tumour that affects young adults aged 15–40 years with an average age of 30 years and a slight male predominance [[1], [2], [3],^9^,^11^].

The most common location of synovial sarcoma is the extremities and particularly the lower limbs in the periarticular regions [4,6,7]. Chest wall and cervicofacial location are rare, the latter is accounting for only 3% of all locations in the body [1,5,12] with predominance in the hypopharynx, parapharyngeal and cervical paravertebral space [1,5,9], and rare cases of maxillary, mandibular, infra-temporal fossa and ethmoidal sinus location have been reported in the literature [2,[8], [9], [10]].

The clinical manifestations of synovial sarcoma of cervicofacial location are non-specific and differ depending on the location of the tumour [1,10]. It most often presents as a painless mass that progressively increases in size over several months until it is large enough to cause pressure on adjacent structures and the appearance of clinical signs, cervical lymphadenopathy may also be found at the time of presentation indicating lymph node extension [1,4,8,10].

Medical imaging (CT and MRI) allows confirmation of the tumoral nature of the lesion and study of its limits and its relationship with neighbouring structures. CT is more interesting for specifying bone erosion and intratumoral calcifications, whereas MRI best specifies the locoregional extension of the tumour, vascular invasion and intratumoral haemorrhage [8,10]. In our case, the tumour was T1-isosignal and T2-hypersignal and was locally advanced by extension to adjacent anatomical regions (orbit and nasal cavity), and the extension workup allowed the fortuitous discovery of the chest wall location.

Macroscopically, synovial sarcoma presents as a rounded, well-limited, pseudo-encapsulated, lobulated or plurinodular mass, sometimes with cystic and haemorrhagic foci and calcifications [10]. Pathologically, synovial sarcoma can be monophasic consisting of epithelial or spindle cells only, or biphasic characterised by the presence of glandular epithelial cells associated with small spindle cells of fibroblastic type, or poorly differentiated consisting of small oval-shaped cells characterised by sparse cytoplasm and a dense nucleus [8,[10], [11], [12]]. Immunohistochemical studies may be necessary in some cases to confirm the diagnosis. Synovial sarcoma expresses Epithelial Membrane Antigen (EMA) and cytokeratins in 90% of cases, CD99 in 60% of cases and S100 protein in 30% of cases [8,10,11]. In our case, the anatomopathological study of the biopsies of the maxillary and anterior chest wall tumours showed a fusocellular tumour proliferation with EMA positivity on immunohistochemistry, which allowed the diagnosis of synovial sarcoma.

Genetically, synovial sarcomas are characterised by a chromosomal translocation t(X;18)(p11.2;q11.2) found in more than 95% of tumours. This results in a fusion between the SYT gene on chromosome 18 with SSX1, SSX2 or SSX4 (very rare cases), which are genes located on the X chromosome. The SYT-SSX2 fusion is usually associated with the monophasic type. The SYT-SSX1 fusion is present in both biphasic and monophasic tumours. The SYT-SSX fusion transcript has a high diagnostic value and can be detected on pathological specimens with a sensitivity of 96% and a specificity of 100% [8,[10], [11], [12]]. The fusion type is a major prognostic factor as the SYT-SSX1 fusion gene is associated with fast-growing, aggressive and metastasising synovial sarcomas: synovial sarcomas with poor prognosis [8].

The therapeutic management of cervicofacial synovial sarcoma is multidisciplinary, including surgeons and oncologists. Large tumour resection with cervical lymph node dissection and adjuvant radiotherapy remains the treatment of choice for non-metastatic localized forms to reduce the risk of local and distant recurrence and to ensure better local control of the tumour [8,11]. The value of adjuvant chemotherapy is not yet well defined, but it appears to theoretically reduce or delay the occurrence of distant metastases [8,10]. For inoperable or metastatic forms, chemotherapy with Doxorubicin and/or Ifosfamide is the first line treatment with a response rate of around 50% [11]. In our case, the presence of two concomitant tumour locations and the locally advanced nature of the maxillary location precluded surgery and the indication for chemotherapy was given.

Local recurrence after tumour removal occurs in 50% of cases, especially in the first two years, lymph node metastases occur in 10–20% of cases and remote metastases occur in 30–42% of cases and are dominated by pulmonary, bone and liver location [7,8,10,11]. In our case there was no lymph node extension of the tumours, and the primary location (maxilla or chest wall) could not be identified so the diagnosis of synchronous dual locations was retained.

Five-year survival varies between 65 and 76% and 10-year survival between 10 and 15% depending on prognostic factors, the most important of which are tumour size, extent of loco-regional and distant invasion, mitotic index, degree of tumour necrosis and feasibility of complete tumour resection [8,11].

## 4 Conclusion

Synovial sarcoma is a very aggressive malignant tumour with a rare cervicofacial location. Its diagnosis is based on a combination of radiological, histological and molecular evidence. Surgical excision associated with irradiation of the tumour bed constitutes the reference treatment for localised and operable tumours. In metastatic or inoperable forms, chemotherapy improves the prognosis of patients. Early diagnosis of this rare entity allows to avoid mutilating surgery and to ensure a better prognosis for the patients. The discovery of a cervico-facial localization of synovial sarcoma imposes the search for distant metastatic or synchronous localizations by the realization of a systematic evaluation of extension by a thoracic CT scan.

## Provenance and peer review

Not commissioned, externally peer reviewed.

## Guarantor

The Guarantor is the one or more people who accept full responsibility for the work and/or the conduct of the study, had access to the data, and controlled the decision to publish.

## Annals of medicine and surgery

The following information is required for submission. Please note that failure to respond to these questions/statements will mean your submission will be returned. If you have nothing to declare in any of these categories then this should be stated.

## Conflicts of interest

Authors of this article have no conflict or competing interests. All of the authors approved the final version of the manuscript

## Sources of funding

The authors declared that this study has received no financial support.

## Ethical approval

Written informed consent was obtained from the patient for publication of this case report and accompanying images. A copy of the written consent is available for review by the Editor-in-Chief of this journal on request.

## Consent

Written informed consent was obtained from the patient for publication of this case report and accompanying images. A copy of the written consent is available for review by the Editor-in-Chief of this journal on request.

## Author contribution

Please specify the contribution of each author to the paper, e.g. study concept or design, data collection, data analysis or interpretation, writing the paper, others, who have contributed in other ways should be listed as contributors.
